# The tail wagging the dog

**DOI:** 10.4103/2152-7806.68335

**Published:** 2010-08-10

**Authors:** Patrick J. Kelly

**Affiliations:** 14 Sutton Place South, New York 10022, USA

An esteemed but embattled colleague has finally decided to step down as Professor and Chairman of the Department of Neurosurgery at a prestigious Medical School and medical center. The issue was alleged “gender bias”: he did not support the promotion of a female faculty member. Perhaps there was “gender bias.” Perhaps she really didn’t deserve promotion. Nonetheless, she filed a lawsuit. The case came to trial and our colleague and his medical center lost – with a 7-figure judgment in favor of the plaintiff.

The case hinged on alleged “sexist remarks” and abusive comments made by the defendant to the plaintiff in the presence of others. Possibly, the alleged remarks were made in bad taste humor – the type of “ribbing” that colleagues give each other. Or maybe the remarks had an edge to them. It doesn’t matter. Haven’t all of us endured snide and even mean-spirited remarks from superiors during our training programs or as junior faculty members? In neurosurgery, one learns to roll with the punches. And if a job becomes intolerable or we do not get what we feel we deserve, we move on to another institution that provides more opportunity. We usually don’t file lawsuits.

Medicine, in general, and neurosurgery in particular, is stressful. Sometimes “humor” is used to relieve the stress and, of course, there are also the occasional barbed remarks that many of us will remember for a lifetime. However, academic promotions should be based on merit – not on threats. A jury of our “peers” (unlikely any of whom has ever set foot in an operating room) has now determined that this should not go on. Everyone should treat everyone else with “respect” and everyone should be given equal treatment – even the marginally capable – especially if they are in a “minority.” Affirmative action in action! But wait a minute – since when are women a minority? In fact, in the USA, at least, women outnumber men.

So what’s the problem? Well, some women have the audacity to try and enter an “old boys club.” And at one time, medicine in general was, in fact, an “old boys’ club.” Spaces in medical schools were limited (as they sill are) and men, it was assumed, would practice medicine for a lifetime. Women, the argument went, would have to drop out from time to time to have babies, spend time with growing children, and tend to marriages and such. It was thought that they would not practice medicine with the same full-time dedication as men who had the luxury of a wife at home keeping the home fires burning while they, the men, labored from dawn to dusk healing the sick.

Some women chose to challenge that, and many were met with overwhelming hostility from male superiors in medical schools and residency programs. But through the force of their personalities, they persevered, finished their training programs and had successful and productive careers. At one time, women in medical schools were a small minority. Now the total number of female applicants exceeds male applicants and women now make up 49% of US medical school graduates. True, some do drop out of medicine to deal with family matters and to pursue other interests. But so do men who tire of 80+ hour workweeks, demanding patients, insurance company bureaucracy, etc., causing them to burn out, drop out, and decide to work for drug companies, write novels, or make a living as expert witnesses, or whatever. Nonetheless, women have had a tough time in the past.

We had six women in my 1966 medical school class of 100 students. I recall one in particular. I’ll call her Barbara (I won’t use her real name because she became quite famous). She was extremely intelligent but had a somewhat abrasive personality that did not make her popular – especially with superiors. In particular, she ran afoul of one of the top faculty surgeons who took an immediate dislike to her. Every day at rounds, this surgeon would viciously berate and embarrass Barbara in front of patients, nurses, residents and her classmates. But that just made Barbara work harder. She studied harder, stayed up late working up patients and writing her reports – only to be ignored and humiliated by the surgeon the next morning. Did she file a lawsuit or report the surgeon to the medical school? No, she did not. She just made herself work even harder.

Finally the surgeon realized that Barbara knew a great deal more medicine than her classmates. And after a while, the surgeon, when informed about a new patient, would by-pass the residents’ and interns’ chart notes and go straight to Barbara’s history, physical exams and write-ups in the patient’s chart in order to find out what was really going on with the patient.

Eventually, the surgeon found out that Barbara wanted to be a surgeon. Guess who helped her get a residency? That surgeon! And when she was finishing her residency, that same surgeon invited her to join his practice as a partner. She had a brilliant career and became one of the most respected and successful surgeons in the city. Was she “discriminated against”? Yes, she was. Was there “gender bias”? No doubt about it! But that just made her stronger.

When I was a neurosurgical resident at Northwestern, I remember Ruth. She was an extremely attractive radiology resident who used to sit in on our morning neuroradiology conferences with Dr. Paul Bucy. Ordinarily the presence of a pretty young lady would put a smile on Bucy’s face and a bounce in his step – as long as she didn’t want to be a doctor. Bucy felt that women had no place in medicine (unless they wanted to be nurses), and he was not circumspect when he had an opinion about something. He made a point of completely ignoring Ruth – until at one of these conferences, a patient’s chest x-ray was put up on the view box.

“That’s a Goddamned Chest x-ray!’” Bucy exclaimed.

“Get a radiologist.”

“Here’s a radiologist,” I piped up, pointing to Ruth.

Like guns on a turret, Bucy’s eyes moved from me to focus on this pretty redhead.

“Patrick,” he said, “That’s not a radiologist; that’s a girl!” (Bucy had a tendency to state the obvious and have it sound like a revelation).

Ruth’s complexion turned the color of her hair. And in the Women’s Lib lingua franca of the early 1970’s, she shot back: “What difference does that make?”

Bucy didn’t answer her, but instead turned to me and said with a sly smile:

“They have stars in their eyes, Sir!” – a sexist remark if there ever was one.

Everyone laughed. And so did Ruth. She then stepped up and competently read the chest x-ray for him.

After that Bucy would occasionally call on Ruth at conferences when one of his residents didn’t know the answer to one of his esoteric questions. She always knew the answer.

“See, Patrick - even a girl knows more than you do!” he’d bluster.

Did Ruth ever report him to the medical school or the “Equal Opportunity Committee” for his “sexist remarks”? Did she file a lawsuit? No, she didn’t. She laughed them off and kept on working. She respected Bucy for who he was and he quickly learned to respect her for her hard work, intelligence and for her determination. Ruth went on to become one of the most respected academic neuro-radiologists in the country.

A few years ago, when I was Chairman of Neurosurgery at NYU, we ranked two female neurosurgical residency applicants as choices Numbers “1” and “2.” They also chose us, and did not disappoint us. In fact, these two young women performed spectacularly. Did they take “abuse”? Yes, from time to time, they did. But so did their male counterparts. That’s part of a neurosurgical program! Neurosurgery is high-pressure, and serious business. As George Tindall once told me: “Pat, we ain’t treatin’ measles.”

This brings me back to the situation I mentioned at the start of this diatribe. Reportedly, the woman doctor in question was forced to work in a “hostile” and “malignant” environment. I’ve visited that institution and I tend to agree; that particular medical center is, indeed, “high pressure” and competitive and, at that institution, that culture seems to breed academic productivity. However, if one cannot tolerate this “unpleasant’ culture, why not simply find another job?

In my opinion, playing the sexual bias card, filing a lawsuit and making this a *cause celebre* has actually hurt the cause of women who wish to enter neurosurgery. Neurosurgical Chairs and Program Directors may now consider women candidates for faculty or residency positions with trepidation. They may see potential sexual bias lawsuits and trouble as the marginally competent can now claim minority or sexual discrimination when they don’t succeed. Defense against these types of lawsuits will require laborious, time-consuming “paper trails” and documentation before an incompetent or lackluster person can be dismissed from a residency or not promoted in an academic position. And even then they could end up as defendants at the mercy of a jury that ponders the relevance of the documentation. Considering all of the bureaucratic concerns that academic chairs must now deal with, who needs to add yet another?

